# Crystal structures of 2,6-bis­[(1*H*-1,2,4-triazol-1-yl)meth­yl]pyridine and 1,1-[pyridine-2,6-diylbis(methyl­ene)]bis­(4-methyl-1*H*-1,2,4-triazol-4-ium) iodide triiodide

**DOI:** 10.1107/S2056989014027881

**Published:** 2015-01-03

**Authors:** Marites A. Guino-o, Matthew J. Folstad, Daron E. Janzen

**Affiliations:** a Chemistry Department, University of St Thomas, Mail OSS 402, Summit Avenue, St Paul, MN 55105-1079, USA; b Department of Chemistry and Biochemistry, St Catherine University, 2004 Randolph Avenue, St Paul, MN 55105, USA

**Keywords:** crystal structure, 1,2,4-triazole, tri­azolium cation, hydrogen bonding

## Abstract

In the structures of the 2,6-bis­(1,2,4-triazoly-3-yl)methyl-substituted pyridine compound, C_11_H_11_N_7_, (I) and the iodide triiodide salt, C_13_H_17_N_7_^2+^·I^−^·I_3_^−^, (II), the dihedral angles between the two triazole rings and the pyridine ring are 66.4 (1) and 74.6 (1)° in (I), and 68.4 (2)° in (II), in which the dication lies across a crystallographic mirror plane. The overall packing structure for (I) is two-dimensional with the layers lying parallel to the (001) plane. In (II), the triiodide anion lies within the mirror plane, occupying the space between the two triazole substituent groups and was found to have minor disorder [occupancy ratio 0.9761 (9):0.0239 (9)]. The overall packing of structure (II) can be described as two-dimensional with the layers stacking parallel to the (001) plane. In the crystal, the predominant inter­molecular inter­actions in (I) and (II) involve the acidic hydrogen atom in the third position of the triazole ring, with either the triazole N-atom acceptor in weak C—H⋯N hydrogen bonds in (I), or with halide counter-ions through C—H⋯I inter­actions, in (II).

## Abstract 2

The predominant inter­molecular inter­actions for triazole rings involve the acidic hydrogen in the third position as shown by the title compound, 2,6-bis­[(1*H*-1,2,4-triazol-1-yl)meth­yl]pyridine, (I), and the salt 1,1′-[pyridine-2,6-diylbis(methyl­ene))bis­(4-methyl-1*H*-1,2,4-triazol-4 -ium] iodide triiodide, (II).

## Chemical context   

1,2,4-Triazole analogs first found applications in the pharmaceutical field as anti­fungal and anti­bacterial agents over 30 years ago. Recent developments are reviewed by Peng *et al.* (2013 ▸). Recently, 1,2,4-triazole rings have been incorporated into ligands used in coordination compounds and polymers (Haasnoot, 2000 ▸; Aromí *et al.*, 2011 ▸; Ouellette *et al.*, 2011 ▸). Related triazolium salts are being used as cations in ionic liquids (Porcar *et al.*, 2013 ▸; Meyer & Strassner, 2011 ▸; Singh *et al.*, 2006 ▸), or as precursors to *N*-heterocyclic carbenes (Lin *et al.*, 2014 ▸; Strassner *et al.*, 2013 ▸; Huynh & Lee, 2013 ▸; Riederer *et al.*, 2011 ▸).
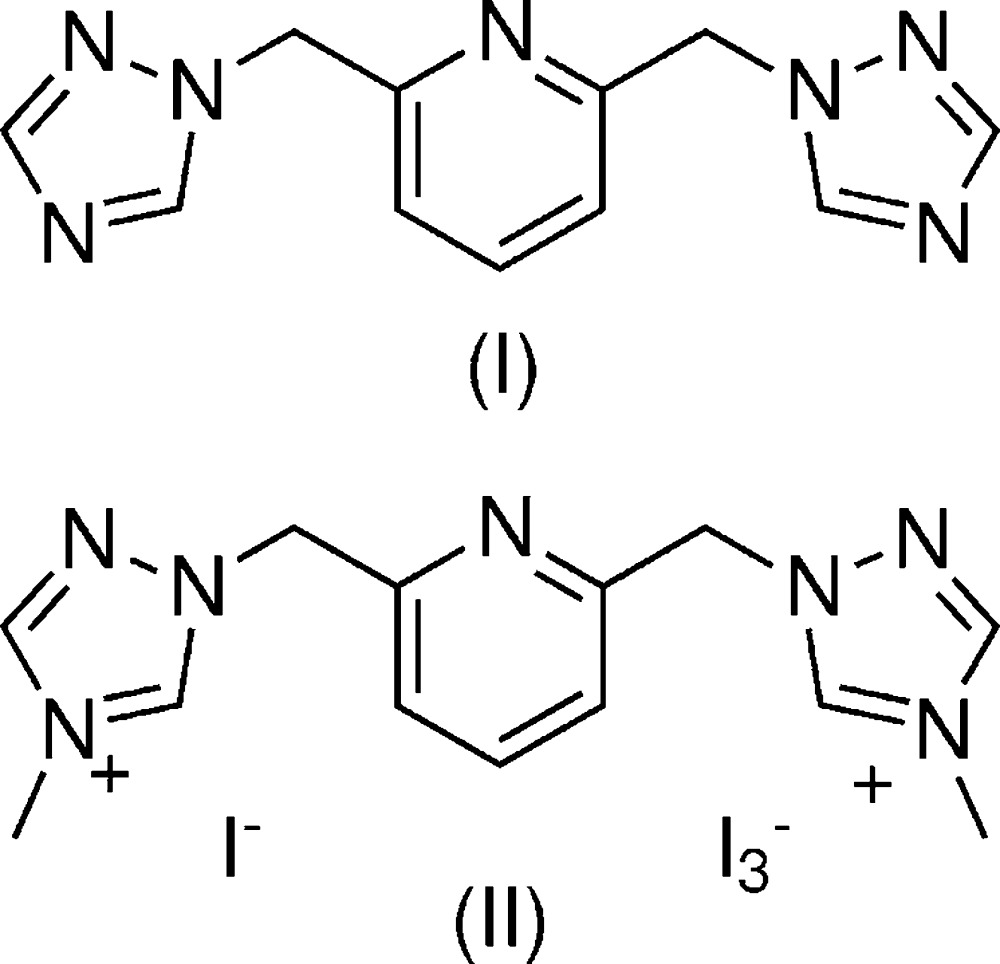


To better understand the suitability of the title compounds for use as ligands for the formation of lanthanide complexes, we became inter­ested in the predominant inter­actions of 1,2,4-triazole rings in the solid state. Herein, we report the structures of 2,6-bis­[(1*H*-1,2,4-triazol-1-yl)meth­yl]pyridine, (I), and 1,1-[pyridine-2,6-diylbis(methyl­ene)]bis­(4-methyl-1*H*-1,2,4-triazol-4-ium) iodide triiodide, (II). The solid-state structures of these compounds by themselves have not been reported, but their structures as ligands in cobalt(II) (Kim *et al.*, 2010 ▸) and palladium(II) complexes (Huynh & Lee, 2013 ▸) are known.

## Structural commentary   

**Figure 1:**
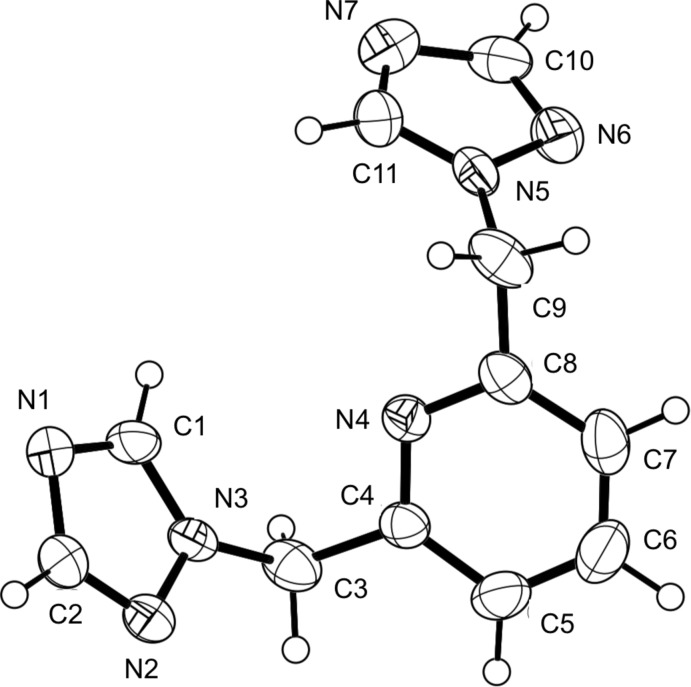
A perspective view of compound (I), showing the atom-numbering scheme. Anisotropic displacement parameters are drawn at the 50% probability level.

Compound (I) crystallizes in the ortho­rhom­bic space group, *Pna*2_1_, with the entire mol­ecule in the asymmetric unit (Fig. 1 ▸). The triazole rings are aromatic with C—C, C—N and N—N bond distances within a range of 1.314 (4) to 1.356 (3) Å. These are twisted above and below the plane of the pyridine ring with dihedral angles between the two triazole rings and the pyridine ring of 66.4 (1) and 74.6 (1)°. The packing structure consists of a stack of triazole mol­ecules with the same handedness translating along the *c*-axis direction. There are no intra­molecular inter­actions due to the inherent steric hindrances within the mol­ecule.

**Figure 2:**
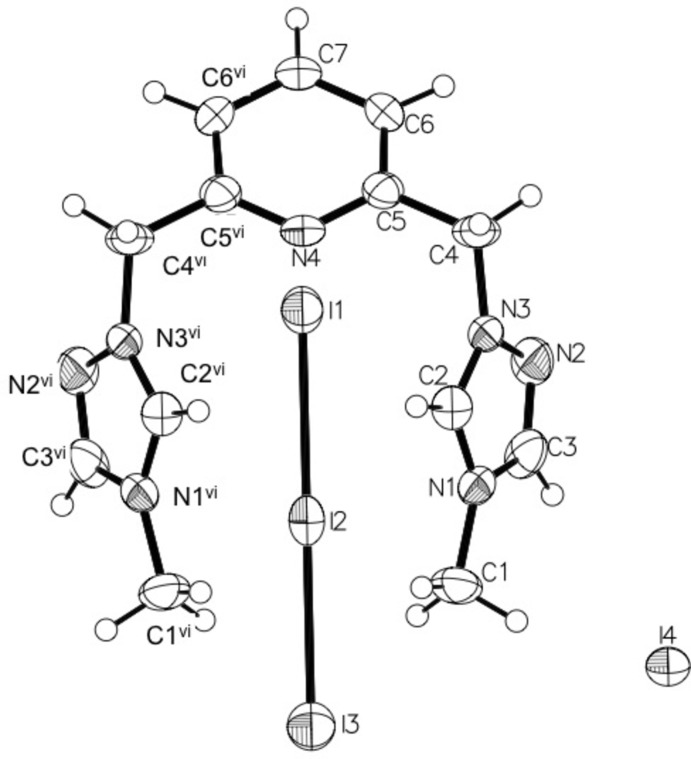
A perspective view of compound (II), showing the atom-numbering scheme, with anisotropic displacement parameters drawn at the 50% probability level. The iodide and triiodide anions lie on crystallographic mirror planes. The minor occupancy component of the disordered triiodide ion is not shown. [Symmetry code: (vi) *x*, −*y* + 1, *z*.]

In contrast, compound (II) consists of a dication of 2,6-bis­[(1*H*-1,2,4-triazol-1-yl)meth­yl]pyridine with methyl groups at the fourth nitro­gen positions of the triazole rings, with mixed triiodide/iodide anions. This compound crystallizes in the space group *C*2/*m* with half of the dication, half of a triiodide (I1—I2—I3) and half of one iodide (I4) in the asymmetric unit (Fig. 2 ▸). The triiodide counter-ion exhibits positional disorder, which was satisfactorily refined with split positions of 0.9761 (9):0.0239 (9), the minor component being I1′—I2′—I3′. Both disorder positions are on the mirror plane and discussions and illustrations relating to this counter-ion are focused on the major occupancy triiodide atom positions. The bond lengths in the triazolium rings indicate significant aromaticity with C—C, C—N, and N—N bond distances in the narrow range of 1.295 (7) to 1.362 (6)Å. The triazole rings are twisted from the plane of the pyridine ring, forming a dihedral angle of 68.4 (2)°. There are no intra­molecular inter­actions.

## Supra­molecular features   

**Figure 3:**
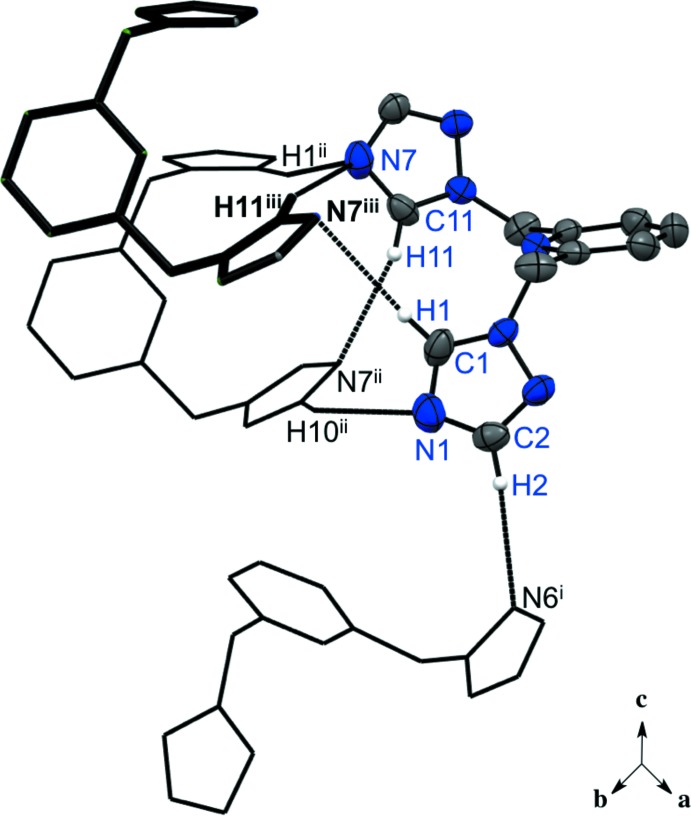
The predominant C—H⋯N hydrogen bonds between triazole rings in one asymmetric unit of compound (I). H atoms not involved in the hydrogen bonding are not shown. For symmetry codes, see Table 1 ▸.

**Figure 4:**
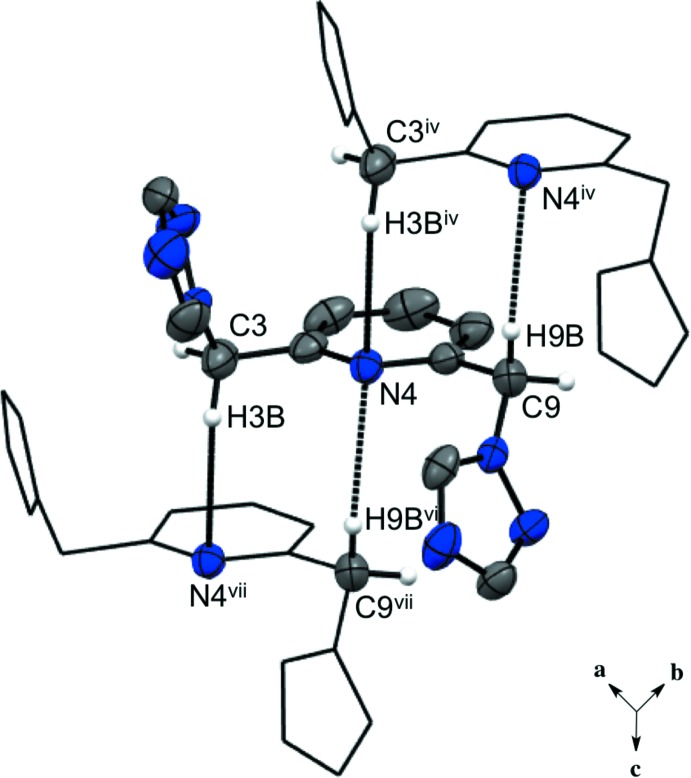
Hydrogen-bond stacking of the pyridine N atoms and the methyl­ene H atoms in compound (I). H atoms not involved in hydrogen bonding are not shown. [Symmetry code: (vii) *x*, *y*, *z* + 1; for other symmetry codes, see Table 1 ▸.]

In compound (I), the predominant inter­molecular inter­actions are the C—H⋯N hydrogen bonds between the acidic hydrogen atoms of the triazole ring and the nitro­gen lone pairs of the neighboring triazole mol­ecule (Table 1 ▸). For one asymmetric unit, there are a total of six hydrogen bonds with three neighboring mol­ecules (Fig. 3 ▸). These hydrogen bonds can be simplified into two categories: *a*) the nitro­gen atoms involved are in the fourth position of the triazole ring (C1—H1⋯N7 and C11—H11⋯N7), and *b*) the nitro­gen atom is in the second position of the ring (C2—H2⋯N6). Pyridine nitro­gen atoms, on the other hand, are involved as acceptors in hydrogen bonds arising from the methyl­ene hydrogen atoms, forming a stack of one mol­ecule on top of the other (Fig. 4 ▸), although no π–π ring inter­actions are present [minimum ring centroid separation, 4.4323 (3) Å]. Additionally, a non-acidic C—H⋯N inter­action is observed between the triazole nitro­gen atom and the *meta*-hydrogen atom of the pyridine ring (C5—H5⋯N2) (Table 1 ▸). The overall packing of structure (I) can be described as layers that lie parallel to (001).

**Figure 5:**
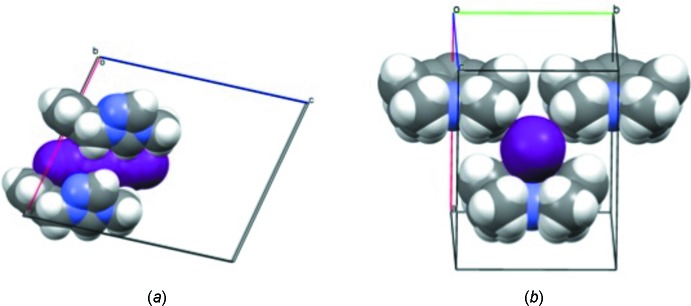
Compound (II) showing the triiodide anion filling up a pore-like groove arrangement built by the triazole dications along (*a*) the *b* axis and (*b*) the *c* axis.

**Figure 6:**
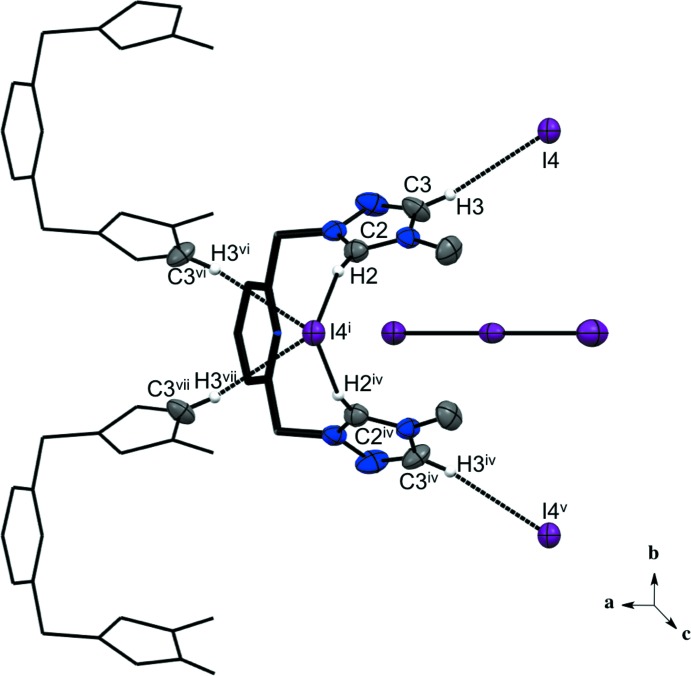
Compound (II) showing the C—H⋯I(iodide) inter­actions. H atoms not involved in hydrogen bonding are not shown. [Symmetry codes: (iv) *x*, −*y* + 1, *z*; (v) *x*, *y* + 1, *z*; (vi) *x* + 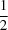, −*y* + 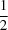, *z*; (vii) *x* + 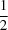, *y* + 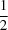, *z*; for other symmetry codes, see Table 2 ▸.]

**Figure 7:**
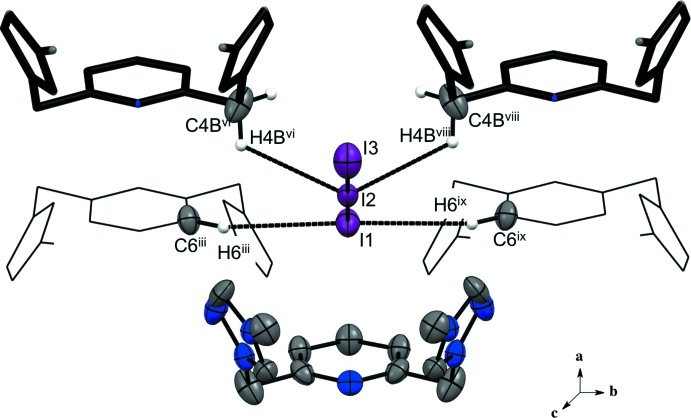
Compound (II) showing the C—H⋯I(triiodide) inter­actions. H atoms not involved in the hydrogen-bonding inter­actions are not shown. [Symmetry codes: (viii) *x* − 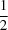, *y* + 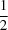, *z*; (ix) −*x* + 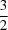, *y* + 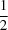, −*z*; for other symmetry codes, see Table 2 ▸ and Fig. 6 ▸.]

In compound (II), when viewed along the *c*-axis, the tri­iodide anion lies on the mirror plane in the middle of the dication-iodide units, filling up a pore-like groove within the structure (Fig. 5 ▸). There are no C—H⋯N inter­actions in compound (II) because the triazole nitro­gen atoms are bonded to the methyl groups. The acidic hydrogen atoms in the triazole ring now prefer to inter­act with the iodide ion. There are four C—H⋯I(iodide) inter­actions per iodide: two from C—H donors from the same dication, and two additional inter­actions from neighboring dication C—H donors (Fig. 6 ▸), (C2—H2⋯I4, C3—H3⋯I4; Table 2 ▸). Meanwhile, the triiodide anion is involved in two C—H⋯I(triiodide) inter­actions with, *a*) the *meta*-hydrogen atoms of the pyridine ring (C6—H6⋯I1), and *b*) the methyl­ene hydrogen atoms (C4—H4*B*⋯I2) (Fig. 7 ▸). The minor occupancy triiodide mol­ecule is not shown, but gives similar inter­actions to those described above for the major component (C6—H6⋯I1′ and C4—H4⋯I2′ as well as C4—H4*A*⋯I1′; Table 2 ▸). The overall packing of structure (II) can be described as two-dimensional with the layers stacking parallel to the (001) plane.

## Database survey   

(1*H*-Imidazol-1-yl){6-[(1*H*-imidazol-1-yl)meth­yl]-2-pyrid­yl}methane (Meng *et al.*, 2005 ▸) is a structure closely related to compound (I). In the solid-state structure, the imidazole nitro­gen atoms prefer to form hydrogen bonds with water mol­ecules in the asymmetric unit, not with the hydrogen atoms of the imidazole ring. In another closely related structure, 2,5-bis­[(1*H*-1,2,4-triazol-1-yl)meth­yl]-1*H*-pyrrole (Lin *et al.*, 2014 ▸), the acidic triazole hydrogen atom also forms C—H⋯N hydrogen-bonding inter­actions similar to those in compound (I).

3-Methyl-1-({6-[(3-methyl-1*H*-imidazol-1-yl)meth­yl]-2-pyrid­yl}meth­yl)-1*H*-imidazole bromide (Nielsen *et al.*, 2002 ▸), a structure closely related to compound (II), crystallizes as a monohydrate. An imidazole hydrogen atom also shows C—H⋯halide(Br) inter­actions, and at the the same time these bromide anions also form hydrogen bonds with the water mol­ecule in the asymmetric unit. Triazolium salt C—H⋯halide inter­actions similar to those shown by compound (II) are also observed in ionic liquids utilizing triazolium cations (Porcar *et al.*, 2013 ▸).

## Synthesis and crystallization   

For the synthesis of compounds (I) and (II), a procedure similar to that reported by Huynh’s group (Huynh & Lee, 2013 ▸) was used. In our attempts, we used the microwave technique for the synthesis of both title compounds but shortened the reaction time for each from 24 hr to roughly 15 min. For (I), 2,6-bis­[(1*H*-1,2,4-triazol-1-yl)meth­yl]pyridine and 1,2,4-triazole (0.0241 mol, 1.665 g) were dissolved in 10–12 mL of aceto­nitrile by stirring. Once these had completely dissolved, K_2_CO_3_ (0.0241 mol, 3.331 g) was added and briefly stirred to deprotonate the triazole. 2,6-Bis(bromo­meth­yl)pyridine (0.011 mol, 2.902 g) was then dissolved separately in 5 mL of aceto­nitrile. The two solutions were then combined in a 10–20 mL microwave vessel and placed in the microwave reactor for 15 min at 403 K, after which the aceto­nitrile was removed *in vacuo*. Compound (I) was isolated through recrystallization utilizing hot di­chloro­methane, producing colorless prismatic crystals suitable for single-crystal X-ray diffraction. Yield 83%. ^1^H NMR (400MHz, CDCl_3_) δ 8.23 (*s*, 2H), 7.98 (*s*, 2H), 7.69 (*t*, 1H), 7.12 (*d*, 2H), 5.44 (*s*, 4H). ^13^C NMR (400 MHz, CDCl_3_) δ 152.5, 144.0, 138.6, 121.8, 54.8.

For (II), 1,1′-[pyridine-2,6-diylbis(methyl­ene)]-bis­(4-methyl-1*H*-1,2,4-triazol-4-ium) iodide and iodo­methane (0.996 mL, 0.016 mol) was added to a 10 mL aceto­nitrile solution of 2,6-bis­[(1*H*-1,2,4-triazol-1-yl)meth­yl]pyridine (0.947 g, 0.004 mol) in a microwave vial. The mixture was placed in the microwave reactor for 10 min at 413 K, after which the aceto­nitrile was removed *in vacuo*. Compound (II) was isolated through recrystallization utilizing isopropyl alcohol layered with hexa­nes, producing brown prismatic crystals suitable for single-crystal X-ray diffraction. Yield 52%. ^1^H NMR (400MHz, DMSO-d6) δ 10.15 (*s*, 2H), 9.17 (*s*, 2H), 7.99 (*t*, 1H), 7.51 (*d*, 2H), 5.76 (*s*, 4H), 3.95 (*s*, 6H). ^13^C NMR (400 MHz, DMSO-*d*6) δ 152.88 (2C), 146.02 (2C), 144.24 (1C), 139.19 (2C), 122.97 (2C), 55.75 (2C), 34.55 (2C), 25.75 (*i*PrOH).

## Refinement   

Crystal data, data collection and structure refinement details are summarized in Table 3 ▸. All hydrogen atoms were placed in calculated positions and allowed to ride on their parent atoms at C—H distances of 0.95 Å for the triazole and the pyridine rings, 0.97 Å for the methyl group and 0.99 Å for the methyl­ene group, with *U*_iso_(H) = 1.2*U*_eq_(C). In compound (II), the triiodide counter-ion showed positional disorder, and the positions were allowed to refine using constraints, introducing split positions of 0.9761 (9):0.0239 (9) (the minor component being I1′—I2′—I3′), with satisfactory refinement.

## Supplementary Material

Crystal structure: contains datablock(s) General, I, II. DOI: 10.1107/S2056989014027881/zs2322sup1.cif

Structure factors: contains datablock(s) I. DOI: 10.1107/S2056989014027881/zs2322Isup2.hkl

Structure factors: contains datablock(s) II. DOI: 10.1107/S2056989014027881/zs2322IIsup3.hkl

Supporting information file. DOI: 10.1107/S2056989014027881/zs2322Isup4.cml

CCDC references: 1040607, 1040608

Additional supporting information:  crystallographic information; 3D view; checkCIF report

## Figures and Tables

**Table 1:** Hydrogen-bond geometry (Å, °) for (I)

*D*—H⋯*A*	*D*—H	H⋯*A*	*D*⋯*A*	*D*—H⋯*A*
C2—H2⋯N6^i^	0.95	2.62	3.547 (4)	166
C11—H11⋯N7^ii^	0.95	2.47	3.381 (4)	161
C1—H1⋯N7^iii^	0.95	2.63	3.551 (4)	164
C9—H9*B*⋯N4^iv^	0.99	2.57	3.419 (5)	144
C5—H5⋯N2^v^	0.95	2.63	3.449 (4)	145

Symmetry codes: (i) 
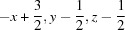
; (ii) 
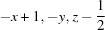
; (iii) 
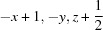
; (iv) 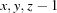; (v) 
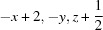
.

**Table 2:** Hydrogen-bond geometry (Å, °) for (II)

*D*—H⋯*A*	*D*—H	H⋯*A*	*D*⋯*A*	*D*—H⋯*A*
C3—H3⋯I4	0.95	3.00	3.844 (5)	149
C2—H2⋯I4^i^	0.95	2.82	3.744 (5)	164
C4—H4*B*⋯I2^ii^	0.99	3.18	3.775 (6)	120
C4—H4*B*⋯I2′^ii^	0.99	3.10	3.766 (15)	126
C6—H6⋯I1^iii^	0.95	3.17	4.097 (5)	166
C6—H6⋯I1′^iii^	0.95	3.00	3.900 (8)	158
C4—H4*A*⋯I1′^iii^	0.99	2.98	3.94 (2)	166

Symmetry codes: (i) 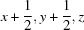; (ii) 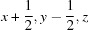; (iii) 
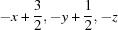
.

**Table 3:** Experimental details

	(I)	(II)
Crystal data		
Chemical formula	C_11_H_11_N_7_	C_13_H_17_N_7_^2+^·I_3_^−^·I^−^
*M*_r_	241.27	778.94
Crystal system, space group	Orthorhombic, *P* *n* *a*2_1_	Monoclinic, *C*2/*m*
Temperature (K)	173	173
*a*, *b*, *c* (Å)	14.465 (3), 18.742 (4), 4.3230 (9)	13.784 (3), 10.010 (3), 16.709 (4)
α, β, γ (°)	90, 90, 90	90, 102.648 (7), 90
*V* (Å^3^)	1172.0 (4)	2249.5 (10)
*Z*	4	4
Radiation type	Mo *K*α	Mo *K*α
μ (mm^−1^)	0.09	5.55
Crystal size (mm)	0.62 × 0.15 × 0.13	0.34 × 0.14 × 0.09

Data collection		
Diffractometer	Rigaku XtaLAB mini	Rigaku XtaLAB mini
Absorption correction	Multi-scan (*REQAB*; Rigaku, 1998 ▸)	Multi-scan (*REQAB*; Rigaku, 1998 ▸)
*T*_min_, *T*_max_	0.779, 0.988	0.322, 0.607
No. of measured, independent and observed [*I* > 2σ(*I*)] reflections	9633, 2381, 1895	11638, 2712, 2303
*R*_int_	0.062	0.037
(sin θ/λ)_max_ (Å^−1^)	0.625	0.649

Refinement		
*R*[*F* ^2^ > 2σ(*F* ^2^)], *wR*(*F* ^2^), *S*	0.047, 0.101, 1.05	0.036, 0.080, 1.11
No. of reflections	2381	2712
No. of parameters	163	126
No. of restraints	1	0
H-atom treatment	H-atom parameters constrained	H-atom parameters constrained
	*w* = 1/[σ^2^(*F*_o_^2^) + (0.042*P*)^2^ + 0.0415*P*] where *P* = (*F*_o_^2^ + 2*F*_c_^2^)/3	*w* = 1/[σ^2^(*F*_o_^2^) + (0.0241*P*)^2^ + 14.4966*P*] where *P* = (*F*_o_^2^ + 2*F*_c_^2^)/3
Δρ_max_, Δρ_min_ (e Å^−3^)	0.13, −0.14	1.09, −1.11

Computer programs: *CrystalClear-SM Expert* (Rigaku, 2011 ▸), *SIR2004* (Burla, *et al.*, 2005 ▸), *SHELXS97* and *SHELXL2013* (Sheldrick, 2008 ▸) and *CrystalStructure* (Rigaku, 2010 ▸).

## supplementary crystallographic information

### (I) 2,6-Bis[(1H-1,2,4-triazol-1-yl)methyl]pyridine Crystal data

**Table:**

C_11_H_11_N_7_	*D*_x_ = 1.367 Mg m^−^^3^
*M**_r_* = 241.27	Mo *K*α radiation, λ = 0.71075 Å
Orthorhombic, *P**n**a*2_1_	Cell parameters from 8485 reflections
*a* = 14.465 (3) Å	θ = 3.0–26.5°
*b* = 18.742 (4) Å	µ = 0.09 mm^−^^1^
*c* = 4.3230 (9) Å	*T* = 173 K
*V* = 1172.0 (4) Å^3^	Prism, colorless
*Z* = 4	0.62 × 0.15 × 0.13 mm
*F*(000) = 504	

### (I) 2,6-Bis[(1H-1,2,4-triazol-1-yl)methyl]pyridine Data collection

**Table:**

Rigaku XtaLAB mini diffractometer	2381 independent reflections
Radiation source: normal-focus sealed tube	1895 reflections with *I* > 2σ(*I*)
Detector resolution: 6.849 pixels mm^-1^	*R*_int_ = 0.062
ω scans	θ_max_ = 26.4°, θ_min_ = 3.0°
Absorption correction: multi-scan (*REQAB*; Rigaku, 1998)	*h* = −18→18
*T*_min_ = 0.779, *T*_max_ = 0.988	*k* = −23→23
9633 measured reflections	*l* = −5→5

### (I) 2,6-Bis[(1H-1,2,4-triazol-1-yl)methyl]pyridine Refinement

**Table:**

Refinement on *F*^2^	1 restraint
Least-squares matrix: full	Hydrogen site location: inferred from neighbouring sites
*R*[*F*^2^ > 2σ(*F*^2^)] = 0.047	H-atom parameters constrained
*wR*(*F*^2^) = 0.101	*w* = 1/[σ^2^(*F*_o_^2^) + (0.042*P*)^2^ + 0.0415*P*] where *P* = (*F*_o_^2^ + 2*F*_c_^2^)/3
*S* = 1.05	(Δ/σ)_max_ < 0.001
2381 reflections	Δρ_max_ = 0.13 e Å^−^^3^
163 parameters	Δρ_min_ = −0.14 e Å^−^^3^

### (I) 2,6-Bis[(1H-1,2,4-triazol-1-yl)methyl]pyridine Special details

**Table:**

Geometry. All e.s.d.'s (except the e.s.d. in the dihedral angle between two l.s. planes) are estimated using the full covariance matrix. The cell e.s.d.'s are taken into account individually in the estimation of e.s.d.'s in distances, angles and torsion angles; correlations between e.s.d.'s in cell parameters are only used when they are defined by crystal symmetry. An approximate (isotropic) treatment of cell e.s.d.'s is used for estimating e.s.d.'s involving l.s. planes.

### (I) 2,6-Bis[(1H-1,2,4-triazol-1-yl)methyl]pyridine Fractional atomic coordinates and isotropic or equivalent isotropic displacement parameters (Å^2^)

**Table:**

	*x*	*y*	*z*	*U*_iso_*/*U*_eq_	
N1	0.74693 (19)	−0.17388 (14)	0.3737 (8)	0.0581 (9)	
N2	0.87942 (16)	−0.11410 (13)	0.4504 (8)	0.0471 (7)	
N3	0.80940 (15)	−0.08386 (12)	0.6125 (7)	0.0344 (6)	
N4	0.75319 (15)	0.06501 (11)	0.4587 (6)	0.0324 (6)	
N5	0.58336 (15)	0.13416 (11)	0.2984 (7)	0.0331 (6)	
N6	0.54335 (16)	0.19034 (12)	0.4433 (7)	0.0410 (7)	
N7	0.46408 (16)	0.08922 (13)	0.5355 (8)	0.0486 (8)	
C1	0.7326 (2)	−0.12001 (18)	0.5624 (11)	0.0527 (10)	
H1	0.6744	−0.1086	0.6513	0.063*	
C2	0.8376 (2)	−0.16807 (16)	0.3142 (9)	0.0466 (8)	
H2	0.8693	−0.2005	0.1832	0.056*	
C3	0.8238 (2)	−0.02053 (16)	0.7995 (9)	0.0479 (8)	
H3A	0.8815	−0.0263	0.9202	0.057*	
H3B	0.7720	−0.0158	0.9478	0.057*	
C4	0.83028 (19)	0.04624 (15)	0.6106 (8)	0.0370 (7)	
C5	0.9105 (2)	0.08642 (18)	0.5924 (10)	0.0503 (9)	
H5	0.9649	0.0713	0.6968	0.060*	
C6	0.9106 (2)	0.14789 (19)	0.4231 (11)	0.0583 (11)	
H6	0.9650	0.1761	0.4103	0.070*	
C7	0.8316 (2)	0.16875 (16)	0.2710 (9)	0.0479 (9)	
H7	0.8299	0.2116	0.1541	0.057*	
C8	0.75449 (19)	0.12512 (14)	0.2939 (8)	0.0344 (7)	
C9	0.6670 (2)	0.14229 (16)	0.1146 (9)	0.0439 (8)	
H9A	0.6706	0.1920	0.0383	0.053*	
H9B	0.6632	0.1105	−0.0677	0.053*	
C10	0.47173 (19)	0.16046 (16)	0.5807 (9)	0.0431 (8)	
H10	0.4289	0.1868	0.7018	0.052*	
C11	0.5357 (2)	0.07513 (15)	0.3578 (9)	0.0429 (9)	
H11	0.5512	0.0290	0.2824	0.052*	

### (I) 2,6-Bis[(1H-1,2,4-triazol-1-yl)methyl]pyridine Atomic displacement parameters (Å^2^)

**Table:**

	*U*^11^	*U*^22^	*U*^33^	*U*^12^	*U*^13^	*U*^23^
N1	0.0518 (18)	0.0445 (15)	0.078 (3)	−0.0075 (13)	0.0027 (18)	0.0032 (18)
N2	0.0368 (14)	0.0460 (16)	0.0585 (19)	0.0077 (12)	0.0104 (15)	−0.0035 (16)
N3	0.0302 (12)	0.0366 (13)	0.0363 (14)	0.0103 (10)	0.0026 (13)	0.0052 (13)
N4	0.0329 (12)	0.0329 (12)	0.0313 (13)	0.0012 (10)	−0.0026 (12)	0.0014 (12)
N5	0.0354 (13)	0.0277 (12)	0.0360 (14)	0.0054 (10)	−0.0040 (13)	0.0027 (13)
N6	0.0381 (14)	0.0332 (13)	0.0517 (17)	0.0066 (11)	0.0028 (14)	−0.0044 (14)
N7	0.0324 (13)	0.0380 (14)	0.075 (2)	0.0003 (11)	−0.0032 (16)	0.0069 (15)
C1	0.0325 (16)	0.0492 (19)	0.076 (3)	0.0049 (15)	0.009 (2)	0.006 (2)
C2	0.060 (2)	0.0330 (16)	0.047 (2)	0.0072 (15)	0.012 (2)	0.0037 (18)
C3	0.059 (2)	0.0491 (19)	0.0351 (18)	0.0175 (16)	−0.0100 (18)	−0.003 (2)
C4	0.0407 (16)	0.0368 (16)	0.0336 (17)	0.0084 (13)	−0.0027 (17)	−0.0110 (16)
C5	0.0336 (16)	0.054 (2)	0.063 (2)	0.0063 (15)	−0.0096 (19)	−0.022 (2)
C6	0.0388 (18)	0.053 (2)	0.084 (3)	−0.0119 (16)	0.010 (2)	−0.017 (2)
C7	0.0506 (19)	0.0322 (16)	0.061 (2)	−0.0024 (14)	0.015 (2)	−0.0041 (19)
C8	0.0358 (15)	0.0317 (15)	0.0358 (16)	0.0037 (12)	0.0070 (16)	−0.0025 (15)
C9	0.0487 (18)	0.0433 (18)	0.0397 (18)	0.0095 (14)	0.0042 (18)	0.0100 (17)
C10	0.0341 (15)	0.0393 (17)	0.056 (2)	0.0056 (13)	0.0041 (18)	−0.0016 (18)
C11	0.0430 (18)	0.0278 (15)	0.058 (3)	0.0016 (13)	−0.0109 (18)	−0.0060 (16)

### (I) 2,6-Bis[(1H-1,2,4-triazol-1-yl)methyl]pyridine Geometric parameters (Å, º)

**Table:**

N1—C1	1.314 (4)	C3—C4	1.497 (4)
N1—C2	1.340 (4)	C3—H3A	0.9900
N2—C2	1.318 (4)	C3—H3B	0.9900
N2—N3	1.356 (3)	C4—C5	1.386 (4)
N3—C1	1.319 (4)	C5—C6	1.365 (5)
N3—C3	1.451 (4)	C5—H5	0.9500
N4—C8	1.333 (4)	C6—C7	1.375 (5)
N4—C4	1.341 (4)	C6—H6	0.9500
N5—C11	1.329 (3)	C7—C8	1.386 (4)
N5—N6	1.355 (3)	C7—H7	0.9500
N5—C9	1.455 (4)	C8—C9	1.519 (4)
N6—C10	1.319 (4)	C9—H9A	0.9900
N7—C11	1.317 (4)	C9—H9B	0.9900
N7—C10	1.354 (4)	C10—H10	0.9500
C1—H1	0.9500	C11—H11	0.9500
C2—H2	0.9500		
			
C1—N1—C2	102.2 (3)	C5—C4—C3	122.5 (3)
C2—N2—N3	102.0 (2)	C6—C5—C4	119.3 (3)
C1—N3—N2	109.3 (3)	C6—C5—H5	120.3
C1—N3—C3	129.2 (3)	C4—C5—H5	120.3
N2—N3—C3	121.5 (2)	C5—C6—C7	119.7 (3)
C8—N4—C4	118.1 (2)	C5—C6—H6	120.1
C11—N5—N6	109.6 (3)	C7—C6—H6	120.1
C11—N5—C9	128.6 (3)	C6—C7—C8	117.8 (3)
N6—N5—C9	121.7 (2)	C6—C7—H7	121.1
C10—N6—N5	102.3 (2)	C8—C7—H7	121.1
C11—N7—C10	102.6 (2)	N4—C8—C7	123.2 (3)
N1—C1—N3	111.3 (3)	N4—C8—C9	116.1 (2)
N1—C1—H1	124.4	C7—C8—C9	120.6 (3)
N3—C1—H1	124.4	N5—C9—C8	113.1 (3)
N2—C2—N1	115.2 (3)	N5—C9—H9A	109.0
N2—C2—H2	122.4	C8—C9—H9A	109.0
N1—C2—H2	122.4	N5—C9—H9B	109.0
N3—C3—C4	112.9 (3)	C8—C9—H9B	109.0
N3—C3—H3A	109.0	H9A—C9—H9B	107.8
C4—C3—H3A	109.0	N6—C10—N7	114.7 (3)
N3—C3—H3B	109.0	N6—C10—H10	122.6
C4—C3—H3B	109.0	N7—C10—H10	122.6
H3A—C3—H3B	107.8	N7—C11—N5	110.7 (3)
N4—C4—C5	121.8 (3)	N7—C11—H11	124.6
N4—C4—C3	115.7 (3)	N5—C11—H11	124.6
			
C2—N2—N3—C1	−0.6 (4)	C3—C4—C5—C6	−178.0 (3)
C2—N2—N3—C3	−179.3 (3)	C4—C5—C6—C7	−0.6 (6)
C11—N5—N6—C10	−0.7 (3)	C5—C6—C7—C8	−0.8 (5)
C9—N5—N6—C10	−179.7 (3)	C4—N4—C8—C7	0.3 (5)
C2—N1—C1—N3	0.3 (4)	C4—N4—C8—C9	177.3 (3)
N2—N3—C1—N1	0.2 (4)	C6—C7—C8—N4	1.0 (5)
C3—N3—C1—N1	178.8 (3)	C6—C7—C8—C9	−175.9 (3)
N3—N2—C2—N1	0.8 (4)	C11—N5—C9—C8	−83.0 (4)
C1—N1—C2—N2	−0.7 (4)	N6—N5—C9—C8	95.8 (3)
C1—N3—C3—C4	−101.9 (4)	N4—C8—C9—N5	46.9 (4)
N2—N3—C3—C4	76.6 (3)	C7—C8—C9—N5	−136.0 (3)
C8—N4—C4—C5	−1.8 (4)	N5—N6—C10—N7	0.6 (4)
C8—N4—C4—C3	178.2 (3)	C11—N7—C10—N6	−0.3 (4)
N3—C3—C4—N4	65.3 (3)	C10—N7—C11—N5	−0.2 (4)
N3—C3—C4—C5	−114.7 (3)	N6—N5—C11—N7	0.6 (4)
N4—C4—C5—C6	2.0 (5)	C9—N5—C11—N7	179.5 (3)

### (I) 2,6-Bis[(1H-1,2,4-triazol-1-yl)methyl]pyridine Hydrogen-bond geometry (Å, º)

**Table:**

*D*—H···*A*	*D*—H	H···*A*	*D*···*A*	*D*—H···*A*
C2—H2···N6^i^	0.95	2.62	3.547 (4)	166
C11—H11···N7^ii^	0.95	2.47	3.381 (4)	161
C1—H1···N7^iii^	0.95	2.63	3.551 (4)	164
C9—H9*B*···N4^iv^	0.99	2.57	3.419 (5)	144
C5—H5···N2^v^	0.95	2.63	3.449 (4)	145

Symmetry codes: (i) −*x*+3/2, *y*−1/2, *z*−1/2; (ii) −*x*+1, −*y*, *z*−1/2; (iii) −*x*+1, −*y*, *z*+1/2; (iv) *x*, *y*, *z*−1; (v) −*x*+2, −*y*, *z*+1/2.

### (II) 1,1-[Pyridine-2,6-diylbis(methylene)]bis(4-methyl-1H-1,2,4-triazol-4-ium) iodide triiodide Crystal data

**Table:**

C_13_H_17_N_7_^2+^·I_3_^−^·I^−^	*F*(000) = 1424
*M**_r_* = 778.94	*D*_x_ = 2.300 Mg m^−^^3^
Monoclinic, *C*2/*m*	Mo *K*α radiation, λ = 0.71075 Å
*a* = 13.784 (3) Å	Cell parameters from 10090 reflections
*b* = 10.010 (3) Å	θ = 3.0–27.5°
*c* = 16.709 (4) Å	µ = 5.55 mm^−^^1^
β = 102.648 (7)°	*T* = 173 K
*V* = 2249.5 (10) Å^3^	Prism, brown
*Z* = 4	0.34 × 0.14 × 0.09 mm

### (II) 1,1-[Pyridine-2,6-diylbis(methylene)]bis(4-methyl-1H-1,2,4-triazol-4-ium) iodide triiodide Data collection

**Table:**

Rigaku XtaLAB mini diffractometer	2712 independent reflections
Radiation source: normal-focus sealed tube	2303 reflections with *I* > 2σ(*I*)
Detector resolution: 6.849 pixels mm^-1^	*R*_int_ = 0.037
ω scans	θ_max_ = 27.5°, θ_min_ = 3.0°
Absorption correction: multi-scan (*REQAB*; Rigaku, 1998)	*h* = −17→17
*T*_min_ = 0.322, *T*_max_ = 0.607	*k* = −12→12
11638 measured reflections	*l* = −21→21

### (II) 1,1-[Pyridine-2,6-diylbis(methylene)]bis(4-methyl-1H-1,2,4-triazol-4-ium) iodide triiodide Refinement

**Table:**

Refinement on *F*^2^	0 restraints
Least-squares matrix: full	Hydrogen site location: inferred from neighbouring sites
*R*[*F*^2^ > 2σ(*F*^2^)] = 0.036	H-atom parameters constrained
*wR*(*F*^2^) = 0.080	*w* = 1/[σ^2^(*F*_o_^2^) + (0.0241*P*)^2^ + 14.4966*P*] where *P* = (*F*_o_^2^ + 2*F*_c_^2^)/3
*S* = 1.11	(Δ/σ)_max_ = 0.001
2712 reflections	Δρ_max_ = 1.09 e Å^−^^3^
126 parameters	Δρ_min_ = −1.11 e Å^−^^3^

### (II) 1,1-[Pyridine-2,6-diylbis(methylene)]bis(4-methyl-1H-1,2,4-triazol-4-ium) iodide triiodide Special details

**Table:**

Geometry. All e.s.d.'s (except the e.s.d. in the dihedral angle between two l.s. planes) are estimated using the full covariance matrix. The cell e.s.d.'s are taken into account individually in the estimation of e.s.d.'s in distances, angles and torsion angles; correlations between e.s.d.'s in cell parameters are only used when they are defined by crystal symmetry. An approximate (isotropic) treatment of cell e.s.d.'s is used for estimating e.s.d.'s involving l.s. planes.

### (II) 1,1-[Pyridine-2,6-diylbis(methylene)]bis(4-methyl-1H-1,2,4-triazol-4-ium) iodide triiodide Fractional atomic coordinates and isotropic or equivalent isotropic displacement parameters (Å^2^)

**Table:**

	*x*	*y*	*z*	*U*_iso_*/*U*_eq_	Occ. (<1)
I1	0.64670 (4)	0.5000	0.08917 (4)	0.04623 (16)	0.9761 (9)
I2	0.60699 (4)	0.5000	0.25579 (3)	0.03549 (14)	0.9761 (9)
I3	0.55437 (5)	0.5000	0.41333 (4)	0.05505 (18)	0.9761 (9)
I1'	0.6681 (18)	0.5000	0.0437 (17)	0.04623 (16)	0.0239 (9)
I2'	0.6331 (15)	0.5000	0.2138 (16)	0.03549 (14)	0.0239 (9)
I3'	0.577 (2)	0.5000	0.3681 (17)	0.05505 (18)	0.0239 (9)
I4	0.62747 (3)	0.0000	0.39551 (3)	0.03401 (13)	
N1	0.8485 (3)	0.2640 (4)	0.3182 (2)	0.0330 (9)	
N2	0.7994 (4)	0.1816 (5)	0.1944 (3)	0.0497 (12)	
N3	0.8876 (3)	0.2471 (4)	0.2021 (2)	0.0338 (9)	
N4	0.9323 (4)	0.5000	0.1270 (3)	0.0324 (12)	
C1	0.8505 (4)	0.2959 (7)	0.4053 (3)	0.0487 (14)	
H1A	0.8496	0.2128	0.4362	0.058*	
H1B	0.7921	0.3498	0.4086	0.058*	
H1C	0.9110	0.3461	0.4288	0.058*	
C2	0.9165 (4)	0.2969 (5)	0.2765 (3)	0.0360 (11)	
H2	0.9752	0.3472	0.2965	0.043*	
C3	0.7785 (4)	0.1942 (6)	0.2659 (3)	0.0443 (13)	
H3	0.7205	0.1583	0.2798	0.053*	
C4	0.9370 (5)	0.2575 (6)	0.1327 (3)	0.0510 (15)	
H4A	0.9171	0.1807	0.0953	0.061*	
H4B	1.0099	0.2534	0.1535	0.061*	
C5	0.9101 (4)	0.3866 (5)	0.0855 (3)	0.0366 (11)	
C6	0.8671 (4)	0.3811 (5)	0.0021 (3)	0.0391 (12)	
H6	0.8522	0.2978	−0.0251	0.047*	
C7	0.8468 (6)	0.5000	−0.0403 (4)	0.0421 (18)	
H7	0.8192	0.5000	−0.0976	0.051*	

### (II) 1,1-[Pyridine-2,6-diylbis(methylene)]bis(4-methyl-1H-1,2,4-triazol-4-ium) iodide triiodide Atomic displacement parameters (Å^2^)

**Table:**

	*U*^11^	*U*^22^	*U*^33^	*U*^12^	*U*^13^	*U*^23^
I1	0.0523 (3)	0.0345 (3)	0.0584 (4)	0.000	0.0263 (3)	0.000
I2	0.0342 (3)	0.0255 (2)	0.0439 (3)	0.000	0.0023 (2)	0.000
I3	0.0775 (4)	0.0464 (3)	0.0400 (3)	0.000	0.0100 (3)	0.000
I1'	0.0523 (3)	0.0345 (3)	0.0584 (4)	0.000	0.0263 (3)	0.000
I2'	0.0342 (3)	0.0255 (2)	0.0439 (3)	0.000	0.0023 (2)	0.000
I3'	0.0775 (4)	0.0464 (3)	0.0400 (3)	0.000	0.0100 (3)	0.000
I4	0.0302 (2)	0.0402 (3)	0.0309 (2)	0.000	0.00506 (17)	0.000
N1	0.040 (2)	0.029 (2)	0.030 (2)	−0.0001 (17)	0.0069 (17)	0.0043 (17)
N2	0.067 (3)	0.038 (3)	0.038 (2)	−0.017 (2)	−0.001 (2)	−0.006 (2)
N3	0.048 (2)	0.027 (2)	0.0266 (19)	0.0058 (19)	0.0082 (17)	0.0040 (17)
N4	0.038 (3)	0.039 (3)	0.022 (3)	0.000	0.010 (2)	0.000
C1	0.063 (4)	0.055 (4)	0.028 (3)	0.007 (3)	0.012 (3)	−0.003 (2)
C2	0.035 (2)	0.034 (3)	0.038 (3)	0.001 (2)	0.006 (2)	0.000 (2)
C3	0.047 (3)	0.040 (3)	0.043 (3)	−0.019 (3)	0.004 (2)	0.002 (2)
C4	0.079 (4)	0.049 (3)	0.032 (3)	0.023 (3)	0.028 (3)	0.011 (3)
C5	0.041 (3)	0.037 (3)	0.036 (3)	0.011 (2)	0.018 (2)	0.005 (2)
C6	0.053 (3)	0.032 (3)	0.035 (3)	0.004 (2)	0.016 (2)	−0.005 (2)
C7	0.059 (5)	0.043 (4)	0.025 (3)	0.000	0.012 (3)	0.000

### (II) 1,1-[Pyridine-2,6-diylbis(methylene)]bis(4-methyl-1H-1,2,4-triazol-4-ium) iodide triiodide Geometric parameters (Å, º)

**Table:**

I1—I2	2.9524 (10)	C1—H1A	0.9800
I2—I3	2.8796 (10)	C1—H1B	0.9800
I1'—I2'	2.98 (3)	C1—H1C	0.9800
I2'—I3'	2.85 (4)	C2—H2	0.9500
N1—C2	1.326 (6)	C3—H3	0.9500
N1—C3	1.347 (6)	C4—C5	1.517 (7)
N1—C1	1.485 (6)	C4—H4A	0.9900
N2—C3	1.295 (7)	C4—H4B	0.9900
N2—N3	1.362 (6)	C5—C6	1.391 (7)
N3—C2	1.317 (6)	C6—C7	1.381 (6)
N3—C4	1.471 (6)	C6—H6	0.9500
N4—C5^i^	1.331 (6)	C7—C6^i^	1.381 (6)
N4—C5	1.331 (6)	C7—H7	0.9500
			
I3—I2—I1	176.19 (2)	N2—C3—N1	112.1 (5)
I3'—I2'—I1'	173.8 (10)	N2—C3—H3	123.9
C2—N1—C3	106.0 (4)	N1—C3—H3	123.9
C2—N1—C1	126.8 (4)	N3—C4—C5	111.6 (4)
C3—N1—C1	127.2 (4)	N3—C4—H4A	109.3
C3—N2—N3	103.9 (4)	C5—C4—H4A	109.3
C2—N3—N2	110.5 (4)	N3—C4—H4B	109.3
C2—N3—C4	128.4 (5)	C5—C4—H4B	109.3
N2—N3—C4	121.1 (4)	H4A—C4—H4B	108.0
C5^i^—N4—C5	117.2 (6)	N4—C5—C6	123.7 (5)
N1—C1—H1A	109.5	N4—C5—C4	117.0 (5)
N1—C1—H1B	109.5	C6—C5—C4	119.3 (5)
H1A—C1—H1B	109.5	C7—C6—C5	118.3 (5)
N1—C1—H1C	109.5	C7—C6—H6	120.9
H1A—C1—H1C	109.5	C5—C6—H6	120.9
H1B—C1—H1C	109.5	C6^i^—C7—C6	118.9 (7)
N3—C2—N1	107.5 (4)	C6^i^—C7—H7	120.5
N3—C2—H2	126.3	C6—C7—H7	120.5
N1—C2—H2	126.3		
			
C3—N2—N3—C2	−0.3 (6)	C2—N3—C4—C5	−84.4 (7)
C3—N2—N3—C4	−179.2 (5)	N2—N3—C4—C5	94.3 (6)
N2—N3—C2—N1	0.5 (6)	C5^i^—N4—C5—C6	1.0 (10)
C4—N3—C2—N1	179.3 (5)	C5^i^—N4—C5—C4	179.1 (4)
C3—N1—C2—N3	−0.4 (6)	N3—C4—C5—N4	59.2 (7)
C1—N1—C2—N3	178.6 (5)	N3—C4—C5—C6	−122.7 (5)
N3—N2—C3—N1	0.0 (6)	N4—C5—C6—C7	0.4 (9)
C2—N1—C3—N2	0.3 (6)	C4—C5—C6—C7	−177.6 (6)
C1—N1—C3—N2	−178.8 (5)	C5—C6—C7—C6^i^	−1.8 (11)

Symmetry code: (i) *x*, −*y*+1, *z*.

### (II) 1,1-[Pyridine-2,6-diylbis(methylene)]bis(4-methyl-1H-1,2,4-triazol-4-ium) iodide triiodide Hydrogen-bond geometry (Å, º)

**Table:**

*D*—H···*A*	*D*—H	H···*A*	*D*···*A*	*D*—H···*A*
C3—H3···I4	0.95	3.00	3.844 (5)	149
C2—H2···I4^ii^	0.95	2.82	3.744 (5)	164
C4—H4*B*···I2^iii^	0.99	3.18	3.775 (6)	120
C4—H4*B*···I2′^iii^	0.99	3.10	3.766 (15)	126
C6—H6···I1^iv^	0.95	3.17	4.097 (5)	166
C6—H6···I1′^iv^	0.95	3.00	3.900 (8)	158
C4—H4*A*···I1′^iv^	0.99	2.98	3.94 (2)	166

Symmetry codes: (ii) *x*+1/2, *y*+1/2, *z*; (iii) *x*+1/2, *y*−1/2, *z*; (iv) −*x*+3/2, −*y*+1/2, −*z*.
